# Correlation of serum levels of Vitamin D_3_ with serum parathormone in nursing mothers and infants 1-6 months’ age from South Punjab, Pakistan

**DOI:** 10.12669/pjms.36.5.2150

**Published:** 2020

**Authors:** Ghulam Mustafa, Muhammad Khalid, Ijaz Ahmed, Muhammad Abu Talib

**Affiliations:** 1 Prof. Dr. Ghulam Mustafa, FCPS (Pediatr Med.)., Department of Pediatric Medicine, The Children’s Hospital & The Institute of Child Health, Multan, Pakistan; 2 Dr. Muhammad Khalid, FCPS (Pediatr Med.), MSc (Epidemiol & Biostatistics)., Department of Pediatric Medicine, The Children’s Hospital & The Institute of Child Health, Multan, Pakistan; 3 Dr. Ijaz Ahmed, FCPS (Pediatr Med.)., Department of Pediatric Medicine, The Children’s Hospital & The Institute of Child Health, Multan, Pakistan; 4 Dr. Muhammad Abu Talib, FCPS (Pediatr Med.)., Department of Pediatric Medicine, The Children’s Hospital & The Institute of Child Health, Multan, Pakistan

**Keywords:** Calcium, Exclusive breastfeeding, Lactating Mother, Parathormone, VD_3_, Vitamin D

## Abstract

**Background and Objective:**

Parathormone (PTH) and serum Vitamin D_3_ (VD_3_) share a complex interplay where increased VD_3_ leads to a negative response on parathormone level. Our objective was to determine the correlation of parathormone (PTH) and Vitamin D_3_ (VD_3_) levels in nursing mothers and infants 1-6 months’ age from South Punjab, Pakistan.

**Methods:**

This study is a secondary data analysis of previously conducted cross sectional study which was conducted at the Department of Pediatric Medicine, Nishtar Medical University, Multan, during August 2010 to June 2011. Study included 67 infants 1-6 months of age and 60 nursing mothers. A venous blood sample was drawn for estimation of VD_3_, calcium, phosphate, alkaline phosphatase, parathormone and albumin. Spearman correlation coefficient was calculated to determine the inverse correlation between PTH and VD_3_ levels.

**Results:**

Mean age (in days) of the infants was 83±53.7 days whereas maternal mean age was 25.53 ± 4.12 years. Median VD_3_ level in infants was 20.90 ng/ml (IQR – 49.5). Median serum PTH levels were 20.90 pg/ml (IQR – 26.17). Median VD_3_ level in nursing mothers was 21.0 ng/ml (IQR 7.2– 43.8). Median maternal serum PTH levels were 20.89 pg/ml (IQR 2.9 – 232.4). Substantial negative relation between VD_3_ and parathormone in infants and mothers was not evident (r = - 0.027, p-value 0.83) and (r = 0.156, p-value 0.23) respectively. A significant positive association between infant and maternal VD_3_ was observed (r_s_ –0.55, p-value < 0.001).

**Conclusion:**

Our study affirms that the customary negative correlation between VD_3_ and parathormone levels does not exist.

## INTRODUCTION

The Parathormone (PTH) and serum Vitamin D_3_ (VD_3_) share a complex interplay where increased calcium and VD_3_ exert a negative feedback on PTH level that has been shown by multiple studies.^1^ Lower level of VD_3_ in the body not only decreases intestinal calcium and phosphorus absorption but also triggers the parathormone secretion through calcium-sensing receptors. This increased concentration of PTH in serum leads to increased bony problems like bone mineralization defects leading to loss of bone & higher chances of bone fractures.^2^ Although this negative relationship of VD_3_ & PTH is recognized but the optimal serum levels of VD_3_ that will keep the PTH levels suppressed is still not clear.^3^

Various studies have differently defined VD_3_ insufficiency in adults using different cut offs of serum VD_3_ as 37.5 nmol/L^4^, 50 nmol/L^5^, or 75 nmol/L.^6^ These cutoffs were determined in relation to fracture risk, intestinal calcium absorption or bone mineral density. Based on increased serum PTH in the wake of low VD_3_ status, the 2008 guidelines by the American Academy of Pediatrics have recommended optimal serum VD_3_ ≥ 50 nmol/L for children.^7^

The optimal level to stop increase in PTH level with decreasing VD_3_ and its inverse relationship does not always hold true. A laboratory database study from Israel included 19, 172 people with complete VD_3_ and PTH tests. A significant negative correlation (r = - 0. 176, p-value < 0.001) was observed. After excluding the patients with hypercalcemia or renal failure; a VD_3_ plateau of 46.2 nmol/L was found that stabilized the serum PTH levels.^8^ However, in a study on 1370 children age 1–6 years, mean reported levels for VD_3_ and PTH were 86 nmol/L & 2.67 pmol/L, respectively. VD_3_ plateau level of 107 nmol/L (P < .001) was identified above which PTH levels were minimized.^3^ However, the studies do not consistently prove this negative relationship between PTH & VD_3_. Choi YJ et al studied 57 VD_3_ deficient infants 1 – 6 months age, 20 of these were evaluated for correlation with serum PTH levels and no significant (Pearson p-value – 0.051) correlation was reported.^9^

Lactating mother-infant pair has been particularly susceptible to VD_3_ deficiency. Maternal VD_3_ status determines the VD_3_ status of exclusively breast-fed infants. In order to evaluate this negative correlation of VD_3_ and PTH we conducted the study on infant – mother in South Punjab, Pakistan.

## METHODS

This study is secondary data analysis of our previous studies that aimed to determine the VD_3_ status and determinants of low VD_3_ in nursing mothers and infants 1 – 6 months of age, which was conducted at Department of Pediatric Medicine (both inpatient and outpatient) and approved by ethical review committee of Nishtar Medical University (ERC#15864-77 dated July 14, 2016). This was a cross sectional study conducted from August 2010 to June 2011 at Multan city of South Punjab, Pakistan. A sample of 67 infants and 60 nursing mothers was enrolled through convenience sampling. The eligibility criteria for Infants included 1-6 months of age who visited for routine immunization, those admitted with acute respiratory illness in the Pediatric Medicine Department and whose parents were willing to participate in the study. The infants with any metabolic illness or congenital anomaly and those who were already on VD_3_ supplementation were excluded from the study. The nursing mothers of these infants were also approached to participate in the study and consenting mothers were enrolled.

### Data collection and laboratory samples

We used two structured questionnaires for data collection from the mothers and infants. The questionnaire for mothers included the demographic data about the family, mother, delivery, diet, daily sun exposure and maternal intake of calcium/ VD_3_ containing vitamins during and after pregnancy. The questionnaire for the infants included the demographic parameters and characteristics including feeding habits that can affect the VD_3_ status. Three milliliters of venous blood were obtained by the standard procedure from both mother & baby in special vials. All the samples were stocked at -2 to -8°C till the analysis were made. VD_3_ estimation was done by FDA approved Abbott Laboratories’ chemiluminescent microparticle immunoassay (CMIA – Abbott Park, IL). We also obtained levels of Ca^+2^, PO_4_^-3^, alkaline phosphatase, parathormone and albumin. Taking reference the current recommendations^10^ the cut-off points for VD_3_ deficiency and VD_3_ insufficiency used were <30 nmol/L and <50 nmol/L, respectively. The optimal level of VD_3_ for infants was taken as >80 nmol/L and >50 nmol/L for nursing mothers.

### Data analysis

The data was analyzed using STATA 12.0. The numerical variables are described as the mean ± standard deviation (SD) & qualitative variables as frequency and percentages. The Spearman correlation coefficient was applied to examine relationship between the PTH & VD_3_ levels.

## RESULTS

Over a period of one year 67 infants 1 – 6 months’ age and 60 lactating mothers (data from 7 mothers was not available) were included in the study. Median VD_3_ level in infants was 20.90 ng/ml (IQR – 49.5). Median serum parathyroid (PTH) levels were 20.90 pg/ml (IQR – 26.17). Mean serum concentration of other biochemical markers included corrected serum calcium (mg/dl) 8.88 ± 0.91, serum phosphate (mg/dl) 5.8 ± 1.03 and serum Alkaline phosphatase (IU/L) 282.4 ± 67. Median VD_3_ level in nursing mothers was 21.0 ng/ml (IQR 7.2– 43.8). Median serum parathyroid (PTH) levels were 20.89 pg/ml (IQR 2.9 – 232.4). Mean serum concentration of corrected serum calcium (mg/dl) was 8.98 ± 0.73, serum phosphate (mg/dl) 4.19 ± 0.97 and serum alkaline phosphatase (IU/L) was 174.68 ± 53.97 (Table-I).

**Table-I:** Biochemical Characteristics of participating infants 1-6 months’ age (n=67) and nursing mothers (n=60).

Biochemistry	Infants		Mothers	
Serum calcium*, mg/dl (mean ± SD)	8.88 ± 0.91		8.98 ± 0.73	
Serum phosphate, mg/dl (mean ± SD)	5.8 ± 1.03		4.19 ± 0.97	
Serum Alkaline phosphatase, IU/L (mean ± SD)	282.4 ± 72.67		174.68 ± 53.97	
Serum Albumin, g/dl (mean ± SD)	3.84 ± 0.36		3.82 ± 0.26	
Serum Vit. D, ng/ml (median, (IQR)	20.90 (49.5)		21.0 (7.2 – 43.8)	

VD_3_ status	n	(%)	n	(%)

Optimal (> 50 ng/ml)	21	(31.3)	00	(00)
Insufficient (30 - < 50 ng/ml)	05	(7.5)	02	(3.3)
Deficient (<30 ng/ml)	41	(61.2)	58	(96.7)
Serum PTH, pg/ml (median, range)	20.90	(26.17)	20.89	(2.9-232.4)

* Corrected serum Calcium proportional to Albumin status.

**Fig.1:**
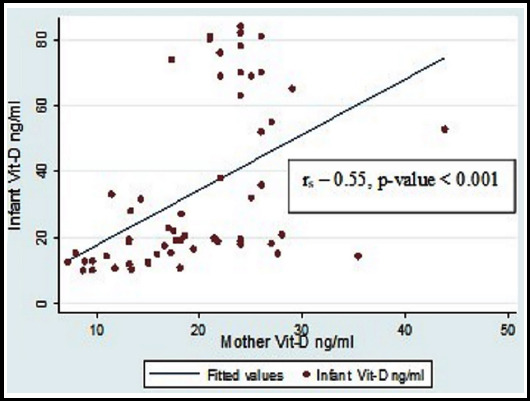
Correlation between Maternal and Infants Vitamin D levels.

No significant negative relation between VD_3_ and parathormone in infants and mothers was evident by Spearman correlation coefficient (r = -0.027, p-value 0.83) and (r = 0.156, p-value 0.23) respectively. Although Pearson correlation coefficient was not significant between serum calcium and PTH levels in infants (r = 0.088, p-value 0.48) but it was positively correlated in nursing mothers (r = 0.422, p-value 0.001). Similar to infant’s results no significant correlation was observed in mothers between serum phosphate (r = -0.066, p-value 0.60 and r = 0.19, p-value 0.15), alkaline phosphatase (r = 0.037, 0.77 and r= -0.14, p-value 0.29) and PTH levels respectively (Table-II). A moderate positive relationship between mother and child VD_3_ status was observed (r_s_ – 0.55, p-value < 0.001) (Fig.1).

**Table-II:** Correlation of Serum Parathyroid (PTH) with Calcium*, Phosphorus, Alkaline phosphatase and VD_3_ levels in participating infants 1-6 months age (n=67) and nursing mothers (n=60).

	Infants		Mothers	

	PTH, pg/ml			

	Correlation^α^ coefficient	p-value	Correlation coefficient	p-value
Serum total calcium*, mg/dl	0.088	0.48	0.422	0.001
Serum phosphate, mg/dl	-0.066	0.60	0.188	0.15
Alkaline phosphatase, IU/L	0.037	0.77	-0.14	0.29
VD_3_^β^, ng/ml	-0.027	0.83	0.156	0.23

* Corrected serum Calcium proportional to Albumin status,

α Pearson correlation coefficient,

β Spearman’s correlation coefficient.

## DISCUSSION

It has been postulated that increasing level of intact parathyroid hormone (iPTH) in response to low VD_3_ level is a better biochemical indicator of deficient or insufficient vitamin D levels. Studies from Pakistan have reported VD_3_ deficiency in healthy adults^11^,^12^, pregnant women^13^, children^14^, adolescents^15^ and have also examined the correlation of VD_3_ with PTH levels^16^. But none of the studies from Pakistan have documented the correlation of VD_3_ and PTH in dyad of nursing mothers and their infants.

A high prevalence of VD_3_ deficiency both in infants and mothers was evident in our previously published studies. Briefly, 67 % (n/N - 45/67) percent of the infants belonged to rural area, 83.6 % (56/67) were having open house, 51 % (n/N - 34/67) of our infants were on exclusive breast feeding and 61 % of the infants (41/67) were VD_3_ deficient (< 30 ng/ml). Mean age of the mothers (in years) was 25.53 ± 4.12. Seventy-three percent (44/60) belonged to rural areas and 86.7 % (52/60) were having open house. Ninety-six percent (58/60) of the mothers were VD_3_ deficient (< 30 ng/ml).^17^,^18^

Maternal serum calcium in our study was comparable to the levels in a study by Wagner et al (8.98 ± 0.7 vs. 9.4 ± 0.4).^19^ Similarly, maternal phosphorus in our study was comparable to the mothers recruited from Charleston, SC (4.19 ± 0.97 vs. 4.14 ± 0.62) but it was higher compared to mothers recruited from Rochester, NY (3.8 ± 0.60). A moderate positive correlation (r_s_ – 0.55, p-value < 0.001) between the maternal and infant VD_3_ was observed. This was concordant with the study by Wagner CL et al showing moderate positive correlation (r – 0.42 – 0.65, p-value < 0.0001) between lactating mother-infant VD_3_ levels.^19^ It was also concordant with the study results by Husain et al who showed a moderate positive relation between VD_3_ levels of mother and infant pair (Pearson coefficient = 0.516, P < 0.001).^20^ This positive relation between Infant & maternal VD_3_ concentrations (r_s_=0.41, P=0.001) was also highlighted by Salameh et al.^21^

Statistically significant negative relation between VD_3_ and parathormone levels in infants (Pearson correlation r - 0.156, p-value 0.23) as well as mothers (Pearson correlation r – 0.16, p-value 0.23) was not demonstrated in our study. This is in line with a number of studies not showing negative correlation of serum PTH and serum VD_3_ levels in infants and mothers. Elsammak et al studied fifty-two women and eighty-seven men blood donors (N = 139) from Saudi Arabia. A statistically significant inverse relationship between PTH & VD_3_ levels was not observed in either of the groups (men, r = 0.35, p=0.75 and women, r = 0.11, p=0.44 respectively).^22^ However, a local study on 50 healthy female nursing staff verified a significantly strong negative relationship between VD_3_ and PTH levels (r-value: -0.781, p-value: <0.001).^23^

However, Husain et al have demonstrated a strong inverse relationship between PTH & VD_3_ (r = -0.66, p-value < 0.001).^20^ Kramer CK et al also described the negative correlation (r = −0.37; P < .0001) in mothers at three months postpartum.^24^ Study by Wagner et al. also highlighted the inverse relationship between VD_3_ & PTH levels in lactating mothers (r = - 0.32) as well as in infants (r = -0.35).^19^ So, the evidence for inverse relationship between VD_3_ & PTH is not conclusive in either way.

### Limitations of the study

One of the study limitations leading to lack of inverse correlation between VD_3_ and PTH in infants and mothers include smaller sample size. This is due to the fact that this was a secondary analysis of data and study was not powered for this objective. Other limitations include variation in measurement method of PTH levels and non-availability of data on serum magnesium levels (hypomagnesemia) that may influence serum PTH levels.

## CONCLUSION

The negative relation between VD_3_ and parathormone levels, as claimed, is not evident in our study. We need to have well-controlled and high-powered studies to elucidate this dilemma.

### Author`s Contribution

**GM** conceived, designed the study and & editing and final approval of the manuscript. Also responsible for the integrity and accuracy of the work

**MK** performed data analysis and interpretation & first draft of manuscript.

**IA** did data collection, manuscript writing and review of final draft.

**MAT** did data collection and manuscript writing and review of final draft review.
